# Prognostic significance of neutrophil-to-lymphocyte ratio in biliary tract cancers: a systematic review and meta-analysis

**DOI:** 10.18632/oncotarget.16143

**Published:** 2017-03-12

**Authors:** Haowen Tang, Wenping Lu, Bingmin Li, Chonghui Li, Yinzhe Xu, Jiahong Dong

**Affiliations:** ^1^ Hospital and Institute of Hepatobiliary Surgery, Chinese PLA General Hospital, Chinese PLA Medical School, Haidian, Beijing, China; ^2^ Chinese PLA Medical School, Haidian, Beijing, China; ^3^ Center for Hepatopancreatobiliary Diseases, Beijing Tsinghua Changgung Hospital, Tsinghua University Medical Center, Changping, Beijing, China

**Keywords:** neutrophil-lymphocyte ratio, biliary tract cancer, overall survival, relapse free survival, prognosis

## Abstract

**Background:**

Inflammation was considered to perform crucial roles in the development and metastasis of malignancies. A heightened neutrophil-lymphocyte ratio has been described to be associated with detrimental survivals in different malignancies. Debate remains over the impact of heightened neutrophil-lymphocyte ratio on survivals in biliary tract cancer. The review evaluated the prognostic value of neutrophil-lymphocyte ratio in biliary tract cancer.

**Methods:**

MEDLINE, the Cochrane Library, EMBASE, and the Chinese SinoMed were systematically searched for relevant articles. Associations between neutrophil-lymphocyte ratio and long-term outcomes were expressed as the hazard ratios and 95% confidence intervals. The odds ratio was utilized to assess the association between neutrophil-lymphocyte ratio and clinicopathological parameters.

**Results:**

Fourteen studies consisting of 3217 patients were analyzed: 1278 (39.73%) in the high pretreatment neutrophil-lymphocyte ratio group and 1939 (60.27%) in the low pretreatment neutrophil-lymphocyte ratio one. The results proved that heightened pretreatment neutrophil-lymphocyte ratio was significantly associated with detrimental overall survival and relapse free survival for biliary tract cancer patients. In addition, elevated neutrophil-lymphocyte ratio was positively correlated with higher carbohydrate antigen 19-9 levels, advanced TNM staging and greater lymph node involvement.

**Conclusion:**

This meta-analysis marked that an increased pretreatment neutrophil-lymphocyte ratio was significantly linked with detrimental long-term outcomes and clinicopathological parameters for patients with biliary tract cancer.

## INTRODUCTION

Biliary tract cancer (BTC) encompasses a heterogeneous collective of malignant neoplasms arising from the epithelium of the whole biliary system, the spectrum of which includes cholangiocarcinoma and gallbladder carcinoma (GBC) [1–3]. As one of the common causes of cancer deaths worldwide, BTC features early lymph node and distant metastases and thus carries poor survival outcomes [4–6]. The recent years have witnessed a gradual increase in incidence and mortality of BTC worldwide [7, 8]. In spite of improvements in therapeutic strategies, the prognosis of BTC still stays disappointing, with a five-year survival rate of 10-20% [9–11] and a median survival of less than one year for unresectable or metastatic BTC [12, 13]. Given this, to find out a dependable prognostic marker for BTC patients is of much essential.

Inflammatory response was considered to perform crucial roles in tumor development and metastasis [14, 15]. Increasing evidence suggested systemic inflammation correlated with unfavorable survivals in a number of cancers [16–19]. Heightened amounts of proinflammatory cytokines and signaling components in patients with malignancies for one aspect might mirror disease activity and body's natural response to the tumor [20], for another aspect would facilitate the formation of new blood vessels and lymphatic vessels [14, 21]. In particular, BTC development and progression have been reported to be closely mediated by chronic biliary inflammation caused by gallstones, chronic hepatitis, etc [22–25]. Thus, inflammatory markers might possibly be used as valuable prognostic predictors for BTC patients. Neutrophil-lymphocyte ratio (NLR) mirroring primary immune response to diverse pathogen-derived or cancer-derived stimuli has been identified as a valuable predictor with prognostic sense, and detrimental long-term outcomes were commonly linked with the elevation of NLR in different tumors [26–31]. Additionally, our previous work has confirmed the prognostic value of NLR elevation in colorectal liver metastasis [31].

Yet, debate remains over the impact of heightened NLR on long-term survival in BTC. Prior researches had produced inconsistent results about the prognostic role of NLR. Lin G and colleagues argued that the elevation of NLR value was incrementally associated with decreased overall survival (OS) for BTC patients [32], while other report failed to identify that prognostic role [33]. In this case, a meta-analysis aiming to review the predictive value of NLR for BTC was performed. Furthermore, the association between NLR values and clinicopathological parameters was analyzed.

## RESULTS

### Study selection and patients characteristics

**Figure 1:**
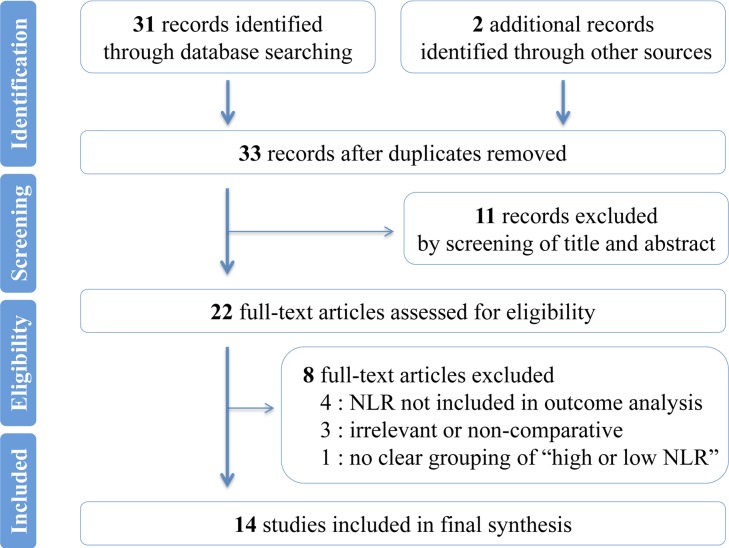
Flowchart of literature search Flowchart showing selection of eligible articles.

Figure 1 shows the process of study selection. A total of 53 references were produced using the outlined search strategy. After application of selection criteria, 14 studies qualified for inclusion in the present meta-analysis [18, 32, 34–45]. Twelve were retrospective studies and two were prospective; the majority (12) of the studies were published in English and two were in Chinese (from the Chinese SinoMed). The studies were carried out in the UK (one study), Romania (one study), Canada (one study), Republic of Korea (one study), Japan (two studies), or China (seven studies) or that were multicenter (the UK and Japan, one study) between 2008 and 2016. Two studies each divided their patients into two cohorts: surgical resection cohort and chemotherapy cohort; one study divided the included patients into the UK cohort and Japan cohort. A total of 3217 patients were analyzed, consisting of 1278 (39.73%) in the high pretreatment NLR group and 1939 (60.27%) in the low pretreatment NLR one. For all the studies, a median of 114.50 patients (range 27-864) were recruited. For the majority of the studies, the median age and median male percentage was 62.77 years (range 53.6-79) and 54.24% (range 33.33-69.23%), respectively. Of all the patients analyzed, 1854 in 11 studies (11 cohorts) received surgical resection and 1363 in five studies (five cohorts) took palliative chemotherapy. The maximum follow-up periods ranged from 48 to 180 months. NLR were all assessed before treatment. Baseline characteristics were presented in Table 1; a summary of main findings were provided in Table 2.

**Table 1:** Characteristics of included studies

First author	Year	Type	Region	Period	Patients characteristics				Tumor type	Treatment (n)	NLR		End point	Maximum follow-up (months)	NOS score
					Total	Age (M±SD)	Male (%)	High NLR (n)			Cut off	Sample time, site			
Lee BS34	2016	R	South Korea	2004-2013	221	62±10	69.23	171	CC	Chemo	5	PT, PB	OS	120	7
Okuno M35	2016	R	Japan	2000-2013	534	66±10	62.92	39	CC	SR	5	PT, PB	OS	78(9-174)¶	7
Lin G29	2016	R	China	1999-2011	102	>50 (n=67)	64.71	43	CC	SR	3	PT, PB	OS RFS	120	7
Li H36	2015	R	China	2011-2014	127	67.50±10.50	56.69	NA	BTC	Chemo(90) &SR(37)	3	PT, PB	OS	29.1(3.9-99.6)†	6
Zhang Y37	2015	R	China	2001-2013	145	63.54±10.46	46.90	83	GBC	SR	1.94	PT, PB	OS	100	6
Chen Q38	2015	R	China	2005-2011	322	58¶	60.25	194	CC	SR	2.49	PT, PB	OS RFS	80	6
Gao HY39,#	2015	R	China	2007-2010	90	53.60(35–87)¶	52.22	16	GBC	SR	5	PT, PB	OS	100	6
Grenader T40	2015	P	The UK &Japan	2002-2008	462	64.30(23-83)†	50.22	134	BTC	Chemo	3	PT, PB	OS	48	6
Iwaku A41	2014	R	Japan	2005-2013	52	79 (52–96)†	59.62	26	BTC	Chemo	4	PT, PB	OS	80	6
McNamara MG42	2014	R	Canada	1987-2012	864	65 (23–93)†	51.39	478	BTC	Chemo(538) &SR(326)	3	PT, PB	OS	14.4(5.6-27.6)†	8
Liu YC43,#	2014	R	China	2002-2011	96	54.80(20–76)¶	66.67	55	CC	SR	2.5	PT, PB	OS RFS	120	7
Wu XS44	2014	R	China	2000-2010	85	≤70 (n=48)	34.12	45	GBC	SR	2.3	PT, PB	OS	16 (2–87)†	6
Dumitrascu T45	2013	R	Romania	1996-2012	90	57.50(24–77)†	57.78	NA	CC	SR	3.3	PT, PB	OS RFS	68(6-143)†	7
Gomez D18	2008	P	The UK	1996-2006	27	57 (32–84)†	33.33	11	CC	SR	5	PT, PB	OS RFS	23(14-72)†	6

NLR: neutrophil-lymphocyte ratio; NOS score: Newcastle-Ottawa Scale score; R: retrospective; P: prospective; CC: cholangiocarcinoma; BTC: biliary tract cancer; GBC: gallbladder carcinoma; Chemo: chemotherapy; SR: surgical resection; PT: pretreatment; PB: peripheral blood; OS: overall survival; RFS: recurrence free survival; NA: not available; &: and. M±SD: mean ±standard deviation; ¶: value is mean with or wihtout range in parenthesis; †: value is median with range in parenthesis; # : studies from the database of the Chinese SinoMed.

**Table 2:** Results of meta-analysis on prognostic significance of NLR in BTCs

	Overall Survival								Recurrence Free Survival					
	No. studies	No. patients	HR	95% CI	I^2^,%	P value for heterogeneity	P value for meta-regression		No. studies	No. patients	HR	95% CI	I^2^,%	P value for heterogeneity
**Overall**	14	3217	1.51	1.36-1.67	14.1	0.3			5	637	1.46	1.18-1.81	41.4	0.15
**Type of cancer**							0.74							
CC	7	1392	1.53	1.31-1.77	0	0.56			5	637	1.46	1.18-1.81	41.4	0.15
GBC	4	1184	1.64	1.08-2.50	68.7	0.02			0	0	-	-	-	-
**Treatment methods**							0.4							
Surgical resection	10	1817	1.49	1.29-1.72	14.2	0.31			5	637	1.46	1.18-1.81	41.4	0.15
Chemotherapy	4	1273	1.53	1.29-1.82	37.4	0.19			0	0	-	-	-	-
**Sample size**							0.28							
≥ 100	8	2777	1.5	1.29-1.73	0.38	0.13			2	424	1.59	0.90-2.80	66.2	0.09
< 100	6	440	1.56	1.29-1.88	0	0.63			3	213	1.46	1.09-1.95	48	0.15
**NLR cutoff**							0.06							
≥ 3	10	2569	1.45	1.28-1.66	24	0.22			3	219	1.79	1.12-2.85	67.1	0.05
< 3	4	648	1.73	1.40-2.14	0	0.87			2	418	1.29	1.03-1.63	0	0.75
**Geographic area**							0.25							
Asian	11	1857	1.67	1.45-1.93	4.7	0.4			3	520	1.43	1.08-1.88	33.4	0.22
Non-Asian	4	1360	1.36	1.20-1.54	0	0.49			2	117	1.65	0.90-3.05	73.9	0.05

NLR: neutrophil-lymphocyte ratio; HR: hazard ratio; CI: confidence interval; CC: cholangiocarcinoma; GBC: gallbladder carcinoma.

### NLR and OS

**Figure 2:**
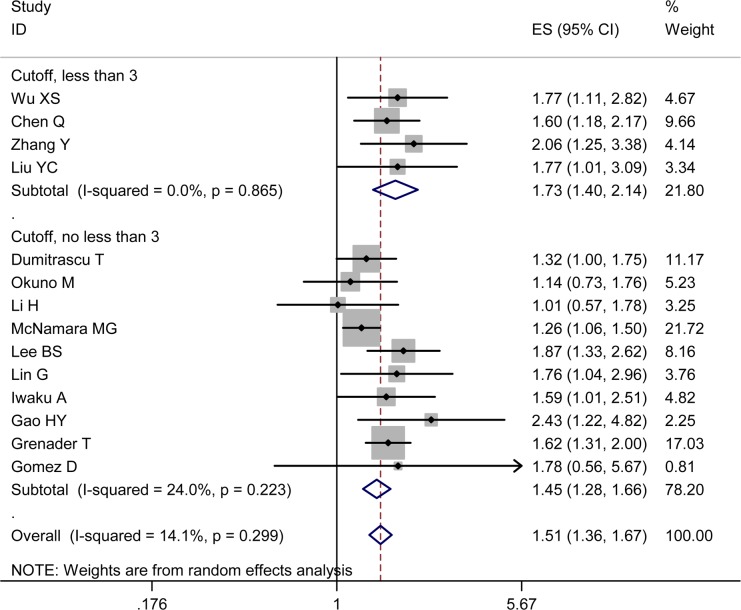
Pooled HR value for OS By subgroups of NLR cutoff value (value ≥ 3 and value < 3) Each square and horizontal bar shows the HR for that trial comparison and corresponding 95% CI, respectively; the size of squares denotes study weight. The diamond represents the pooled HR (random effect model); the center of diamond represents the HR with the extremities denoting the 95% CI.

All the 14 studies investigated the relationship between NLR and OS in BTC. The synthesized HR for high pretreatment NLR group was 1.51 (95% CI 1.36-1.67) in comparison with the low pretreatment NLR group (heterogeneity: I^2^ 14.10%, *P* = 0.30), which implied that an elevation in pretreatment NLR significantly correlated with unfavorable OS for BTC patients (Figure 2).

### NLR and RFS

**Figure 3:**
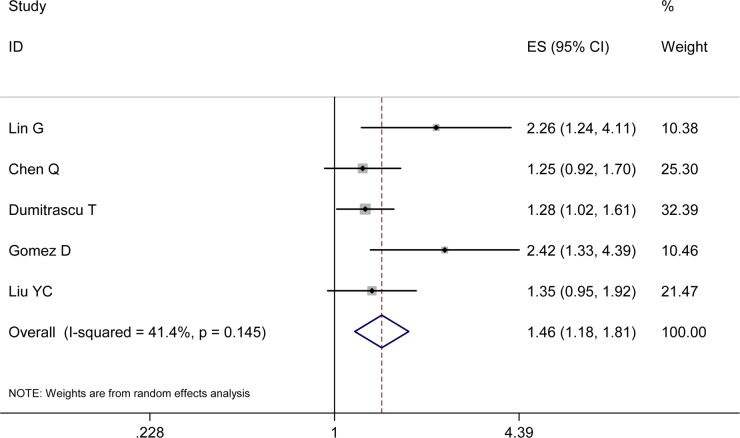
Results of the meta-analysis on pooled HR values for RFS Each square and horizontal bar shows the HR for that trial comparison and corresponding 95% CI, respectively; the size of squares denotes study weight. The diamond represents the pooled HR (random effect model); the center of diamond represents the HR with the extremities denoting the 95% CI.

Five studies documented the relationship between NLR and RFS. The synthesized HR for the high pretreatment NLR group was of statistical sense (HR 1.46, 95% CI 1.18-1.81; heterogeneity: I^2^ 41.40%, *P* = 0.15), which signified that BTC patients with high pretreatment NLR carried detrimental RFS (Figure 3).

### Subgroup analyses and meta-regression

According to the five predefined parameters, we planned subgroup analyses in an attempt to explore the relationship between NLR and prognosis. As to OS, results of all ten subgroup analyses consistently demonstrated that patients with low pretreatment NLR carried survival superiority. With respect to RFS, subgroup analyses were performed based on eight predefined parameters (type of cancer: CC, predominant treatment arm: surgical resection, sample size: size ≥ 100 and size < 100, NLR cutoff value: value ≥ 3 and value < 3, and geographic area: Asian and non-Asian). Subgroup analyses of GBC and chemotherapy were not performed for lack of relevant or enough data. As a result, similar results were achieved, except that no substantial differences in RFS were detected between high NLR and low NLR groups in the subgroups with a sample size ≥ 100 (HR 1.59, 95% CI 0.90-2.80) and with patients from non-Asian geographic area (HR 1.65, 95% CI 0.90-3.05). Results of the meta-regression analysis for OS (type of cancer, *P* = 0.74; predominant treatment arm, *P* = 0.71; sample size, *P* = 0.28; NLR cutoff value, *P* = 0.06; geographic area, *P* = 0.25) confirmed that the heterogeneity among studies was slight. However, meta-regression analysis for RFS was not conducted for limited studies for inclusion, which was best suitable for analyzing at least ten studies. A summary of the above-mentioned results were provided in Table 2.

### Association between NLR and clinicopathological parameters

**Figure 4:**
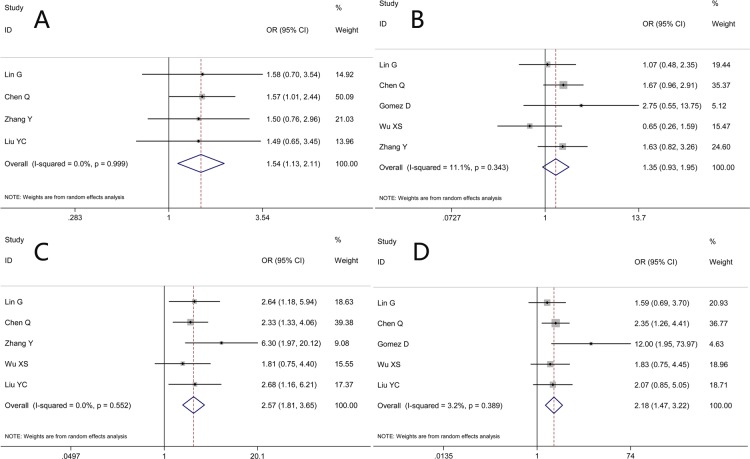
Results of studies on the associations between elevated NLR and clinicopathological parameters Figure 4A implies the association between NLR and pretreatment carbohydrate antigen 19-9 (CA199) level, the result demonstrating a significant correlation between elevated pretreatment NLR and higher CA199 level with no between-study heterogeneity. Figure 4B implies the association between pretreatment NLR and tumor differentiation, the result demonstrating that associations between high NLR values and poorer differentiation were with marginal significance. Figure 4C implies the association between pretreatment NLR and TNM staging, indicating a propensity of heightened pretreatment NLR towards an advanced TNM staging. Figure 4D implies the association pretreatment NLR and lymph node involvement; a similar propensity of lymph node involvement favoring elevated NLR group was presented with slight between-study heterogeneity.

Association between NLR and carbohydrate antigen 19-9 (CA199) level were documented in four studies. The pooled result demonstrated a significant correlation between elevated pretreatment NLR and higher CA199 level (Figure 4A, OR 1.54, 95% CI 1.13-2.11; heterogeneity: I^2^ 0.00%, *P* = 0.99).

As to the correlation between NLR and tumor differentiation, pooled analysis of five studies produced an OR of 1.36 favoring high NLR group (heterogeneity: I^2^ 11.10%, *P* = 0.34); however, corresponding 95% CI ranged from 0.97 to 1.91 (Figure 4B), which indicated that correlations between high NLR and poorer differentiation were with marginal significance.

With regards to TNM staging, relevant parameters from five studies were pooled. The combined OR of 2.60 (Figure 4C, 95% CI 1.84-3.68; heterogeneity: I^2^ 0.00%, *P* = 0.55) illustrated a propensity of heightened pretreatment NLR towards an advanced TNM staging.

In addition, information on NLR and lymph node involvement in BTC patients were extracted from five studies. A similar propensity of lymph node involvement favoring elevated NLR group was also presented (Figure 4D, OR 2.20, 95% CI 1.50-3.21) with slight between-study heterogeneity (I^2^ 3.20%, *P* = 0.39).

### Analysis of sensitivity and test for publication bias

**Figure 5:**
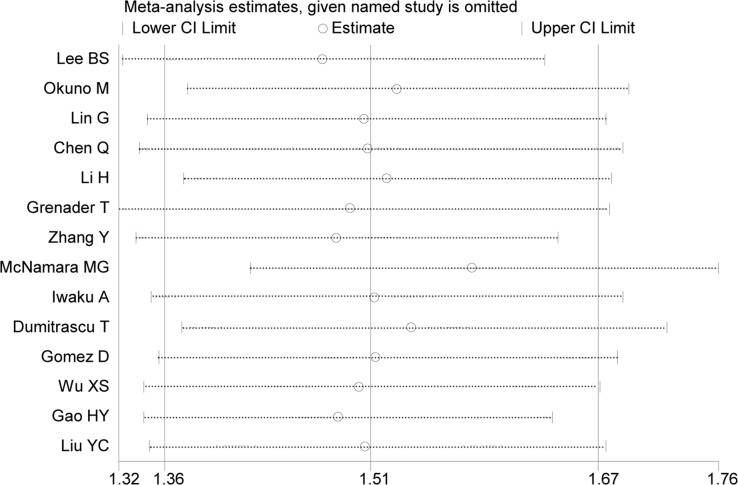
Result of sensitivity analysis for OS The middle vertical line denotes the pooled HR, and the two vertical lines describe 95% CI. The middle small circle and two ends of the dotted lines indicates the pooled HR and 95% CI, respectively, when the study on the left was deleted at a time.

**Figure 6:**
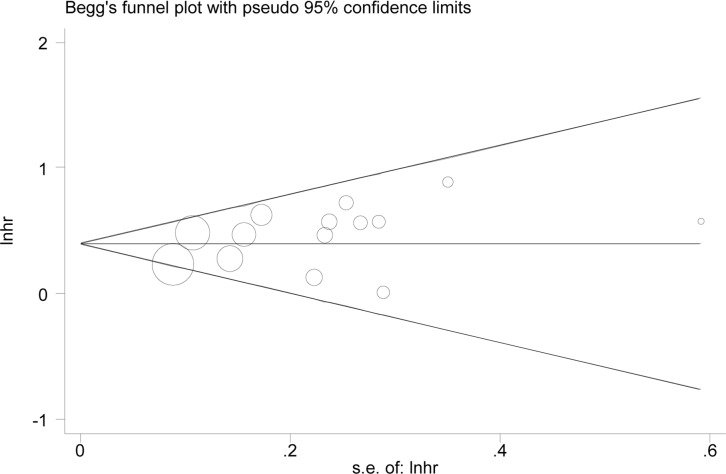
Begg's funnel plot to evaluate OS Begg's funnel plot demonstrated a symmetric distribution showing no existence of evident publication bias for OS.

The results of sensitivity analyses identified no substantial changes in HR values, with a range from 1.47 to 1.58 of HRs in OS (Figure 5) and a range from 1.29 to 1.47 of HRs in RFS. The result of Eegg's test (*P* = 0.14) showed no existence of evident publication bias, with Begg's funnel plot demonstrated a symmetric distribution (Figure 6).

## DISCUSSION

The prognostic significance of NLR has been described in certain malignancies recently. Yet, predictive value of NLR in BTC stays controversial. The present study, to our best knowledge, serves as the first meta-analysis exploring the correlation between NLR and long-term outcomes as well as clinicopathological parameters in BTC patients. The results proved that heightened pretreatment NLR was significantly associated with detrimental OS and RFS for BTC patients. The heterogeneity among studies was slight,. Commensurate results were obtained in most of subgroup analyses: Consistent findings were identified by sensitivity analysis. Also, significant associations between NLR and CA199 level, TNM staging and lymph node involvement were detected.

The definite mechanisms for the relationship between NLR elevation and survival inferiorities for BTC patients have not been fully studied. And the following aspects might explain the correlation.

In our study, elevated NLR was found to be positively correlated with higher CA199 levels, advanced TNM staging and greater lymph node involvement. Regarding BTC, such characteristics has been proven to be linked with tumor invasiveness and metastasis [46], and considered as independent predictors for poor survival outcome. Herein, these findings could partially reflect the prognostic value of increased NLR. In addition, excessive or prolonged chronic inflammation tended to trigger cancerogenesis of normal cells [15]. Earlier studies have shown that inflammation, by stimulating angiogenesis and causing localized immunosuppression, will promote and accelerate the formation of a suitable microenvironment where the survival, expansion, accumulation of successive mutations, and epigenetic changes of premalignant cells could be facilitated [47]. As above mentioned, most of the risk factors for BTC including gallstones and chronic hepatitis might cause such inflammatory response and induce a chronic tissue damage/inflammation mechanism that closely mediates BTC development and progression [22–24]. Recent researches have reflected that NLR signifies the balance between immunosurveillance (function of anti-tumor immune) and tumor-promoting inflammation (activation of pro-tumor inflammatory pathway). Neutrophils are recognized as the primary source of vascular endothelial growth factor, which serves as an angiogenic mediator involved in tumor angiogenesis, and thusly promoted the development and proliferation of malignancy [48–50]. At the meantime, neutrophil elevation prompts the secretion of cytokines and chemokines, thus expediting tumor proliferation [15, 19]. Inhibition of interleukin-6 has been reported to specifically pose a growth-inhibition effect on cholangiocarcinoma cell line, and interleukin-6 overexpression will lead to activation of AKT and anti-apoptotic protein myeloid cell leukemia-1 [51]. Comparatively, lymphocytes, as an indispensable mediator in anti-tumor activity, will cause cytotoxic cell death and cytokine production to eliminate tumor cells [52]. It has been described by Ropponen KM that heightened amounts of tumor-infiltrating lymphocytes independently help foresee favorable survival in colorectal cancer [53]; lymphocyte reduction denoted a suppressed or weakened antitumor immune response [54]. Furthermore, it was illustrated *in vitro* that peripheral blood neutrophil elevation retarded the cytolytic activity of lymphocytes and natural killer cells to tumor cells [55]. Thus, rise of NLR, caused by either a heightened amount of neutrophil or a decreased level of lymphocyte, symbolized the potential inhibition of body's immunosurveillance and antitumor immune response. To sum up, the above-mentioned aspects might be responsible for the result that BTC patients with high NLR featured survival inferiority. This was in close agreement with the findings from McNamara MG who documented that increased pretreatment NLR ascribed to excessive inflammatory response and weakened anti-tumor immunity was significantly linked with poor prognosis in BTC patients. Median survival durations for high NLR patients (NLR ≥ 3) and low NLR patients (NLR < 3) were 12 months and 21.6 months, respectively [42].

Subgroup analyses suggested that prognostic values of elevated NLR for unfavorable OS were obtained in accordance with all ten predefined parameters. Notably, although parallel findings of subgroup estimations for RFS were identified by the majority of predefined parameters, negative prognostic significances of elevated NLR were obtained in the subgroups with a sample size ≥ 100 (HR 1.59, 95% CI 0.90-2.80) and with patients from non-Asian geographic area (HR 1.65, 95% CI 0.90-3.05). Such unmatched performances could be in part explained by the small sizes of included studies for analyses (only two studies for each) and obvious between-study heterogeneities in the pooled analyses (I^2^ equals to 66.20% and 73.90%, respectively).

In addition, NLR took advantages of fast access, widespread availability and economical reproducibility over other laboratory markers. As was shown by our result, a heightened NLR strongly correlated with worse OS and RFS in patients with BTC. Hence, NLR can be regularly surveyed as a prognostic marker for patients with BTC, irrespective of therapeutic arms (surgical resection or chemotherapy) and geographic area (Asian and non-Asian).

There were three main strengths in the present review. (1) The present study, to our best knowledge, serves as the first meta-analysis exploring the correlation between NLR and long-term outcomes as well as clinicopathological parameters in BTC patients. (2) Using relatively strict study selection criteria, a substantial retrospective cohort of 3217 patients were included and analyzed. (3) Heterogeneity in our meta-analysis was slight (I^2^ 14.10%, *P* = 0.30), and commensurate results were accordingly obtained by both subgroup and sensitivity analyses.

Despite of the strengths aforementioned, this review carried the following limitations. The primary limitation was that the majority of the included cohorts were retrospective, thus inevitably influencing the precision of the results. Besides, variations in NLR cutoff values (ranged from 1.94 to 5) possibly exacerbated heterogeneity and bias, which restricted its general application. Furthermore, certain HRs and corresponding 95% CIs (two for both OS and RFS) were retrieved from univariate analyses because of unavailability of these values from multivariate analyses and absences of the authors’ replies. The consistency, accuracy and statistical power of results might be impaired. Finally, the size of the studies included for the pooled estimate of RFS, particularly the subgroup analysis according to predefined parameters, was rather small, which was more susceptible to certain biases.

## CONCLUSIONS

In conclusion, the present review indicated that an increased pretreatment NLR was significantly linked with detrimental long-term outcomes (OS and RFS) and clinicopathological parameters for patients with BTC. Thus, NLR, as a cost-effective and widely available marker with prognostic value, could be regularly surveyed in BTC patients receiving either surgical resection or chemotherapy. Further multicenter and high-quality studies will be needed to support the argument and find out the best NLR cutoff.

## MATERIALS AND METHODS

The meta-analysis was performed in compliance with the Meta-analysis of Observational Studies in Epidemiology group (MOOSE) guidelines [56].

### Study identification

For this review, four electronic databases (MEDLINE (*via* PubMed), the Cochrane Library, EMBASE, and the Chinese SinoMed (http://www.sinomed.ac.cn/zh/)) were systematically searched from the initiation of the databases until September 2016. No language restriction was applied to the search strategy. Search terms (medical subject headings or keywords) included: “Bile Duct Neoplasms,” “Cholangiocarcinoma,” “Common Bile Duct Neoplasms,” “Bile Duct Cancer,” “Biliary Tract Cancer or Neoplasms,” “Gallbladder Cancer, Neoplasm or Carcinoma,” “Cholecyst Cancer, Neoplasm or Carcinoma,” “Neutrophil-lymphocyte (Ratio),” “Neutrophil (to) Lymphocyte (Ratio),” and “NLR.” Furthermore, reference lists of retrieved studies were searched manually. In the presence of repeated papers by authors on the same data set, the latest or most informative was included. The most recent search was done on September 29, 2016. The following selection (inclusion and exclusion) criteria were established in the present meta-analysis.

Inclusion criteria were as follows (i) patient were diagnosed as having BTC (cholangiocarcinoma or GBC) by pathology. (ii) inclusion of pretreatment NLR as a component in final outcome analysis. (iii) clear statement of NLR cutoff. A study fulfilling all three inclusion criteria was regarded eligible to be included. And exclusion criteria were (i) researches focusing on cell assay or animal model. (ii) reviews, letters, comments, case reports and editorials (iii) overlapping or duplicate publications. (iiii) hazard ratio (HR) investigating correlation between pretreatment NLR and long-term survivals unavailable or unobtainable. A study matching any of the four exclusion criteria was ruled out.

### Data abstraction

Two reporters (HW T and BM L) separately abstracted following information from each eligible study using a standardized sheet. Predefined parameters were as follows: study identifier (first author and year of publication); essential study data (study type and region, recruitment period, type of cancer, predominant treatment arms, total number of subjects, the site and time of sampling, NLR cutoff, patient number with increased NLR, monitoring endpoint, HR and confidence interval (CI), and follow-up period); and baseline characteristics of subjects (patient age and male patient percentage, etc.). In the absences of HR, CI, or other vital data from a study, the corresponding author of the study was inquired by email. When failing to get a response, we utilized the methods described by Tierney to digitize and derive the relevant survival estimate [57].

### Definition

Cholangiocarcinoma included three broad categories according to anatomical location (intrahepatic, hilar and extrahepatic) [4]. NLR was calculated from the differential count by dividing the serum absolute neutrophil count by the serum absolute lymphocyte count in peripheral blood [58]. OS referred to the interval between medical interventions and death or the final observation for surviving patients. Relapse free survival (RFS) was calculated from the time of curative treatment until the detection of tumor relapse (recurrence). Tumor differentiation was rigorously stratified according to the British Society of Gastroenterology guidelines on the management of BTC as well/moderated and poorly differentiation. Tumor grade was described using the TNM staging system (American Joint Committee on Cancer 7^th^ edn) as grade I/II and III/IV. Newcastle-Ottawa Scale (NOS) was utilized for the quality assessment for articles included by examining three aspects (method of patient selection, comparability of the study groups, and assessment of outcomes reported). Articles achieving six or more stars were acknowledged to be of good quality. *P* < 0.05 was acknowledged statistically significant.

### Outcomes comparison and statistical analysis

Associations between NLR and long-term outcomes (OS and RFS) were expressed as the HR and a corresponding 95% CI. An HR above one represented a survival benefit favoring low pretreatment NLR group (reference group). The odds ratio (OR) was utilized as the effective value to assess the association between NLR values and clinicopathological parameters.

Analyses were conducted using STATA statistical software (version 12.0, Stata Corporation, College Station, TX, USA). Cochrane's Q and I^2^ tests were performed to evaluate heterogeneity among studies. Random effect models were used because of heterogeneities among studies. Begg's funnel plot and Egger's test were used to examine the publication bias. Sensitivity analysis was conducted in which one study was deleted at a time. An attempt to explain heterogeneity was made using subgroup analyses and meta-regression in accordance with predefined parameters: type of cancer (cholangiocarcinoma and GBC), predominant treatment arm (surgical resection and chemotherapy), sample size (size ≥ 100 and size < 100), NLR cutoff value (value ≥ 3 and value < 3), and geographic area (Asian and non-Asian).

## Abbreviations

NLR: neutrophil-lymphocyte ratio
NOS score: Newcastle-Ottawa Scale score
BTC: biliary tract cancer
GBC: gallbladder carcinoma
OS: overall survival
RFS: relapse free survival
CA199: carbohydrate antigen 19-9.
